# Dimethylcyclosiloxanes in Mobile Smart Terminal Devices: Concentrations, Distributions, Profiles, and Environmental Emissions

**DOI:** 10.3390/toxics12040287

**Published:** 2024-04-13

**Authors:** Yuanna Xing, Yiming Ge, Shaoyou Lu, Tao Yang, Xianzhi Peng

**Affiliations:** 1 Guangzhou Institute of Geochemistry, Chinese Academy of Sciences, Guangzhou 510640, China; xingyuanna18@sina.com (Y.X.); yangtao21@mails.ucas.ac.cn (T.Y.); 2 University of Chinese Academy of Sciences, Beijing 100049, China; 3 School of Public Health (Shenzhen), Shenzhen Campus of SunYat-sen University, Shenzhen 518107, China; geym5@mail2.sysu.edu.cn (Y.G.); lushy23@mail.sysu.edu.cn (S.L.)

**Keywords:** dimethylcyclosiloxanes, mobile smart terminal devices, environmental emission, silicone polymers

## Abstract

Dimethylcyclosiloxanes (DMCs) are utilized as vital monomers in the synthesis of organosilicon compounds, integral to the manufacture of mobile smart terminal devices. Toxicological studies have revealed potential endocrine-disrupting activity, reproductive toxicity, neurotoxicity, and other toxicities of the DMCs. This study investigated the concentrations and composition profiles of seven DMCs, including hexamethylcyclotrisiloxane (D3), octamethylcyclotetrasiloxane (D4), decamethylcyclopentasiloxane (D5), dodecamethylcyclohexasiloxane (D6), and tetradecamethylcycloheptasiloxane (D7), hexadecamethylcyclooctasiloxane (D8), and octadecamethylcyclononasiloxane (D9) in three types of mobile smart terminal device components (silicone rubber, adhesive, and plastics). Environmental emissions of DMCs from silicone rubber materials were also estimated to improve the recognition of their potential fate within the environment. D5–D9 were widely present in silicone rubber and adhesives with detection rates ranging from 91–95.5% and 50–100%, respectively, while D3 and D4 were more frequently detected in plastics, both showing a detection rate of 61.1%. Silicone rubber had the highest total DMCs (∑7DMCs) and a concentration of 802.2 mg/kg, which were dominated by D7, D8, and D9. DMCs detected in adhesives were dominated by D4, D5, and D6. The estimated emission of ∑DMCs released into the environment in China from silicone rubber used in mobile smart terminal devices exceeds 5000 tons per year. Further studies are needed on the presence of DMCs in various commodities and environmental media to assess their ecological and human health impacts, as well as the toxicological effects of D7–D9 for the appropriate regulation of these chemicals.

## 1. Introduction

Dimethylcyclosiloxanes (DMCs) are a class of cyclic compounds with siloxanes as the main chain, characterized by low water solubility, high lipophilicity, and elevated vapor pressure [1,2]. Due to their thermal stability, lubricating properties, and diminished surface tension [3], these compounds play a pivotal role as fundamental raw materials and intermediates to synthesize silicone polymers. Notable derivatives include silicone oils, silicone resins, and silicone rubbers, contributing indispensably to a range of products, encompassing personal care products (PCPs), cosmetics, textiles, automobiles, construction, consumer electronics, baby products, and food [4,5,6,7,8,9]. According to the statistics released by the United States Environmental Protection Agency (EPA) in 2022, the total octamethylcyclotetrasiloxane (D4) production ranged from 750 million to 1 billion pounds during the year 2015. Based on a database from the European Chemicals Agency (ECHA), D4, decamethylcyclopentasiloxane (D5), and dodecamethylcyclohexasiloxane (D6) are annually produced and imported into the European Economic Area in volumes of up to 1 million tons, 100,000 tons, and 10,000 tons, respectively, defined by EPA as high-production volume chemicals.

During industrial production, packaging, and product utilization, the release of DMCs into the environment is an inevitable consequence, facilitated by processes of volatilization and sewage discharge. Consequently, DMCs are widely present in various environmental components, such as air [2,10], waters [11,12], soil [13], sediments [14], and house dust [15,16], as well as organisms [17,18].

Organisms are exposed to DMCs primarily via inhalation, ingestion, or epidermal contact [19,20,21]. However, a review reported the accumulation and potential toxic effects of DMCs in aquatic organisms, owing to their high organic carbon partition coefficients as well as their marked lipophilicity and inherent resistance to biodegradation [22]. It is generally acknowledged that D4, beyond its characterization as an endocrine disruptor with potential estrogenic and antiandrogenic activity [23], could induce hepatic drug-metabolizing enzymes, liver enlargement, inflammatory responses, and hepatocyte death in rats [24,25]. Apart from this, both D4 and D5 are reproductively toxic to rats [26,27], with the former damaging the nervous system of mice offspring [28]. Notably, crayfish exposed to D6 exhibited altered antioxidant capacity and gene expression [29]. Toxicology studies also shed light on the possible link between DMCs and cancer. For instance, D4 and D5 act as dopamine agonists and inhibit prolactin secretion, thereby engendering prolonged stimulation of the endometrium and precipitating endometrial adenomas in rats [30,31]. DMCs may also induce breast cancer by facilitating damage to DNA [32]. A recent epidemiologic study established an association between volatile DMCs and non-alcoholic fatty liver disease in humans [33].

Considering the environmental persistence, bioconcentration, and potential health hazards of DMCs [34,35], researchers have paid mounting concern to these substances. In 2018, the ECHA emphasized the significance of DMCs by officially designating D4, D5, and D6 as Substances of Very High Concern (SVHC) with PBT (Persistent, Bioaccumulative, And Toxic) properties [36]. In June, 2023, ECHA published a restriction proposal to list D4, D5, and D6 under the Stockholm Convention on Persistent Organic Pollutants (POPs), proposing further restrictions to mitigate the risks to humans and the environment [37]. Although the deleterious effects of hexamethylcyclotrisiloxane (D3), D4, D5, and D6 have been the subjects of extensive research, with a consensus emerging on the imperative to limit their use to minimize their hazards, there is little research data on the toxicological effects of tetradecamethylcycloheptasiloxane (D7), hexadecamethylcyclooctasiloxane (D8), and octadecamethylcyclononasiloxane (D9), and their impacts on the ecological environment and human health.

Polysiloxanes synthesized with the participation of DMCs are the majority in use, accounting for more than 90% of the total use of organosilicon. In 2022, China’s polysiloxane capacity and production reached 2.31 million tons and 1.91 million tons, respectively, occupying more than a half of the world [38]. Silicone rubber, silicone adhesives, and silicone copolymer-modified plastics are widely used silicone polymers. Silicone rubber facilitates the manufacture of complex-shaped products renowned for its exceptional waterproof performance, elasticity, and insulation [39,40], and is widely added to mobile smart terminal devices, especially materials that come into contact with human skin, such as remote device control buttons, cell phone cases, smartwatch bands, ear caps, or neck bands for Bluetooth headsets. Compared to ordinary polycarbonate (PC), silicone copolymer-modified PC boasts enhanced properties including flexibility, hydrolysis resistance, corrosion resistance, oxidation resistance, and flame retardancy, and are widely used as shells, covers, and structural components for mobile smart terminal devices [41,42]. The organic silicone adhesive, in addition to bonding and sealing, also has a moisture-proof, temperature, insulation, and shockproof role, accordingly enjoying widespread favor in the potting of various electronic devices [42,43,44,45]. In the intricate process of producing mobile smart terminal products with silicone polymers, DMCs can remain in the products due to incomplete reactions or eliminations, subsequently releasing to the environment during processes such as e-waste dismantling [46] and thermal treatment [47]. Particularly concerning is the substantial disposal of silicone rubber as household waste due to its low price and high frequency of replacement. In China, the annual demand for silicone rubber is as high as 2 million tons, with the predicted requirement for silicone adhesive in 2025 reaching 1350 tons [48]. Furthermore, China manages a diverse array of solid household refuse, encompassing metals, glass, organic matter, textiles, and cigarette butts [49]. However, most of the mobile smart terminal devices are not effectively handled [50]. Therefore, the environmental risk of DMCs caused by releasing from mobile smart terminal device components cannot be ignored.

DMCs have become a class of emerging contaminants due to their wide presence in human plasma and fat samples [51,52,53]. Previous studies have provided important insights into DMCs in PCPs, cosmetics, baby products, and food contact materials. Despite the extensive use of mobile smart terminal devices, a dearth of research exists regarding the content of DMCs within them and their potential environmental risks have been reported, notably concerning the presence of D7–D9. In addition, due to the volatility and long atmospheric half-life exceeding 10 days, the major environmental burdens of D3, D4, and D5 are present mainly in the air [54,55]. Consequently, the calculation of environmental emissions for DMCs with similar structures can provide clues for further understanding of the fate of DMCs in the environment.

In this context, this study investigated the occurrence and profiles of seven DMCs (D3–D9) in silicone rubber, silicone adhesive, and silicone copolymerized modified plastics currently produced and used in mobile smart terminal devices in China. The amount of ∑DMCs (the sum of D3–D9) released into the environment was also estimated.

## 2. Materials and Methods

### 2.1. Chemicals and Reagents

D3 (purity ≥ 99.9%) and D4 (purity ≥ 99.9%) were purchased from TMStandard. D5 (purity ≥ 98.4%), D6 (purity ≥ 97.4%), and D7 (purity ≥ 99.1%) were purchased from Anpel Resi Standard Technical Service. D8 (purity ≥ 98.2%), D9 (purity ≥ 96.0%), and acetone with chromatographic purity was purchased from Guangzhou Chemical Reagent Factory. An overview of the analyzed dimethylcyclosiloxanes is provided in Table 1.

### 2.2. Sample Collection and Preparation

A total of 52 mobile smart terminal product material samples were purchased from official channels and online platforms in China, including silicone rubber samples (*n* = 22), plastic samples (*n* = 18), and adhesive samples (*n* = 12). The silicone rubber samples were subcategorized into ear caps (*n* = 9), watch bands (*n* = 11), and case covers (*n* = 2).

Adhesive samples were prepared in advance. Five grams of adhesive monomer was spread on a 5 cm × 50 cm tin foil and then placed for three days before sample extraction. Silicone rubber and plastic samples do not require special sample preparation before extraction.

The silicone rubber, plastic, and cured adhesive samples were cut into small pieces of 0.5 cm × 0.5 cm samples. One gram of the cut sample was placed in a glass tube and 10.0 mL of acetone was added. Then, the samples were ultrasonically extracted for 2 h. After resting to room temperature, the extract was transferred to a 15 mL tube and centrifuged at 20,000 rpm for 3 min. Then, the supernatant was passed through a 0.22 µm PTFE filter membrane (Jinteng Experimental Equipment, Tianjin, China.) and subjected to instrumental analysis.

### 2.3. Instrumental Analysis

DMCs were determined and quantified by a QP2020NX gas chromatography-mass spectrometer (GC-MS, Shimadzu Corporation, Japan) set to electron impact ionization (EI) mode. The target analytes were separated on an SH-VMS quartz capillary column (60 m × 0.32 mm × 1.8 µm). Helium (purity > 99.999%) was used as a carrier gas, with a flow rate of 1.0 mL/min. The injection volume was 1 µL with splitless mode and the inlet temperature was set to 230 °C. A programmed heating regimen was applied to the column temperature, ranging from 40 °C to 230 °C at a rate of 10 °C/min, and held for 15 min. The source temperature was 230 °C and the ionization voltage was 70 eV, with a transmission line temperature of 230 °C. Quantification was operated by selected ion monitoring with a solvent delay time of 4.5 min. Optimized instrumental parameters are presented in the Supporting Information (Supplementary Table S1).

### 2.4. Quality Assurance and Quality Control

Target analyte standards at different concentrations (1 mg/L, 10 mg/L, and 100 mg/L) were spiked into samples of silicone rubber, plastics, and adhesive to determine recoveries. The recoveries ranged from 82.3% to 107% with the relative standard deviations (RSDs) of 1.5% to 4.0%, collectively demonstrating the stability and reliability of the method. The standard curves of DMCs ranged from 0.1 mg/L to 5 mg/L, and the regression coefficients (*R*^2^) were ≥0.999. The limits of quantitation (LOQ), defined as ten times of signal to noise, were all 1 mg/kg (Table S2).

### 2.5. Environmental Emissions of DMCs from Silicone Rubber

In this study, the estimated amount of DMCs released into the environment in China from silicone rubber used in mobile smart terminal devices was calculated according to the following equations:

(1) $$M=M_{a}+M_{b}+M_{c}$$

where *M* (kg) is the total weight of ∑DMCs (D3, D4, D5, D6, D7, D8, and D9) in all silicone rubber samples; *M_a_*, *M_b_* and *M_c_* (kg) represent the total weight of ∑DMCs in the ear caps, watch bands, and case covers, respectively.

(2) $$M_{x}=\frac{\sum_{1}^{n}\left(c\times m\right)}{n}\times Q_{s}$$

where *M_x_* (kg) represents *M_a_*, *M_b_*, or *M_c_*; *c* (mg/kg) is the concentration of ∑DMCs in the silicone rubber samples; *m* (g) is the weight of the selected sample, with the weights of the ear caps in this study being three pairs; *n* is the number of ear caps, watch bands and case covers analyzed in the present study, which is 9, 11, and 2, respectively; *Q_s_* (Pcs) is the marketed quantity of ear caps, watch bands, and case covers shipment quantities, which are expressed as the number of smart headphones (78.98 million), smart bracelet watches (56.88 million), and smartphones (351 million) shipped, respectively [56].

We performed the Kruskal–Wallis H test or Mann–Whitney U test for differences in the concentrations of DMCs in different samples since the determined DMCs concentrations were under non-normality. Differences were considered statistically significant if *p* < 0.05. Half of LOQ was replaced by the level below LOQ when calculating the average and median concentrations. Data processing was carried out using IBM SPSS 26.0.

## 3. Results and Discussion

### 3.1. Detection Rates and Concentrations of DMCs

A summary of detection rates and concentrations of seven DMCs (D3–D9) in the components of different mobile smart terminal devices were presented in Table 2. Overall, 86.5% of the samples analyzed (*n* = 52) exhibited the presence of at least one detectable DMC. The median concentrations of DCMs varied considerably, ranging from 0.50 mg/kg (D3) to 11.0 mg/kg (D6).

Specifically, the DMCs were detected in 95.5% of silicone rubber samples, slightly below the 100% detection rate observed in silicone nipples from USA [9]. The detection rate of DMCs in adhesive samples was 100%, while that in plastic samples was 66.7%. The high detection rates of DMCs in this study reflected that the DMCs were prevalently applied to silicone polymers. However, individual DMCs exhibited notable variability among the three product samples. D5 was the most frequently detected DMC in all samples, consistent with the results of Canadian cosmetic products [7]. In contrast, D3 and D4 had lower detection rates, which were 34.6% and 57.7%, respectively. Moreover, the median concentrations of D3 and D4 were approximately one to two orders of magnitude lower than those of other DMCs, potentially attributed to their lower boiling points of 134 °C and 175–176 °C, respectively. Nonetheless, D3 and D4 were the most highly detected DMCs in plastics, which could be explained by the fact that DMCs with higher molecular weights, such as D6 and D7, are more commonly used in rubber products [57].

**Figure 1:**
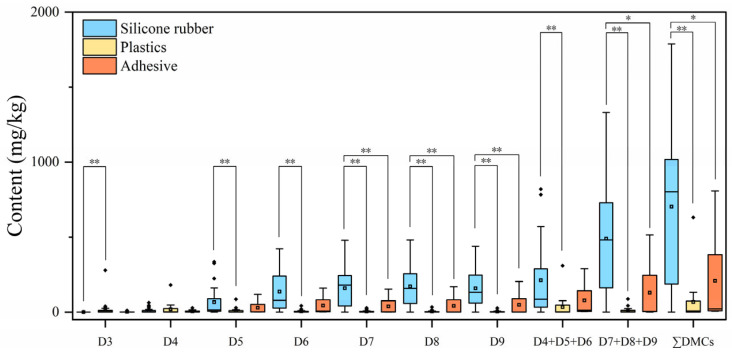
Concentrations (mg/kg) of DMCs in silicone rubber, plastic, and adhesive samples, respectively. The line in the box represents the median value; the bottom and the top of each box represent the 5th and 95th percentiles, respectively; * represents *p* < 0.05, and ** represents *p* < 0.01.

The median concentration of ∑DMCs in silicone rubber samples was 802.2 mg/kg, followed by 21.5 mg/kg in adhesive samples, and 8.25 mg/kg in plastic samples. There is a trend in silicone rubber samples where higher median concentrations of DMCs were associated with increased molecular weights of DMCs. However, D3 and D4 were predominant in plastic samples, and D6 dominated in adhesive samples. As depicted in Figure 1, among the three types of samples, the concentrations of individual DMCs were found to be the highest in silicone rubber, except for D4, which was the highest in plastic samples (*p* < 0.05). One plausible reason for this was that DMCs were typically incorporated in small amounts as additives during the manufacture of certain plastics [58], resulting in lower residue levels in plastics compared to silicone rubber and adhesive samples, where DCMs were added as raw materials.

As shown in Table 3, the concentrations of D4 (<LOQ-63 mg/kg), D5 (<LOQ-335 mg/kg), and D6 (<LOQ-422 mg/kg) in the silicone rubbers in this study were comparable to those in the US silicone nipples [9], but were two to three orders of magnitude higher than those in Chinese soft rubber toys (median: 0.062–0.101 μg/g) and pacifiers (median: 0.107–1.46 μg/g) [58]. The concentrations of D4 (median: 2.15 mg/kg), D5 (median: 0.50 mg/kg), and D6 (median: 0.50 mg/kg) in the plastics in this study were also much higher than those in Chinese hard toys (median: <0.7 × 10^−3^ μg/g). Several studies have reported the concentrations of DMCs in other types of products, including baking molds (mean: 284–1325 mg/kg), Chinese personal care products (median: 0.06–1.70 μg/g), and European cosmetics and personal care products (median: 0.011–25.7 mg/g wet weight) [4,5,59]. In general, the amount of DMCs in silicone products made from different materials can vary greatly, even from the same material. But a common thread lies in the high vapor pressure of DMCs, leading to their continuous release into the environment during use. A higher baking temperature has been documented to increase the release of small molecular weight DMCs from baking molds [60]. The aforementioned mobile smart terminal products, such as personal computers, Bluetooth headsets, and cell phones, are normally used indoors, and the prolonged use of electronic products will heat up, which may be responsible for the increase in the volatilization of DMCs. While this conjecture requires further experimental validation, it raises concerns about the potential negative impacts on human health from long-term skin contact or inhalation of substantial amounts of DMCs.

### 3.2. Composition Profiles of DMCs

**Figure 2:**
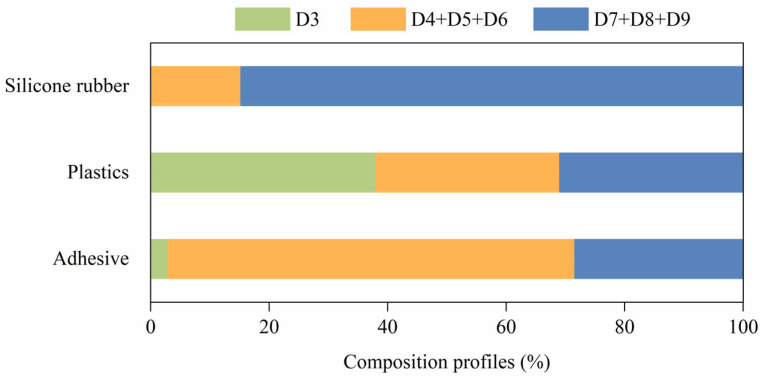
Composition profiles of DMCs in silicone rubber, plastics, and adhesive samples, respectively (%).

The compositional profiles of D3, D4+D5+D6, and D7+D8+D9 in silicone rubber, plastic, and adhesive samples are presented in Figure 2. Among the three products, the profiles of DMCs are quite different, indicating that the types and contents of DMCs required for different materials can vary dramatically. The compositional percentage in the adhesive was in the order of D4+D5+D6 (68.8%) > D7+D8+D9 (28.4%) > D3 (2.8%), while the proportions of components in the plastic were about the same. The sum concentration of D7, D8, and D9 in silicone rubber samples accounted for more than 80% of the ∑DMCs, which was five times higher than that of D4+D5+D6 (481.5 m g/kg vs. 86.0 mg/kg). This finding is similar to the results observed in bakeware, where the sum of the average concentrations of D7, D8, and D9 is approximately threefold higher than that of D4, D5, and D6 combined (3717 mg/kg vs. 1542 mg/kg) [5]. Among the analyzed DMCs, D3 exhibits the lowest boiling point of 134 °C, rendering it prone to volatilization during the production and use of the products, resulting in lower concentrations in the silicone rubber and adhesive samples. However, it remains to be verified whether the atmospheric concentration of D3 is the most minimal. Generally, D4, D5, and D6 are widely utilized as raw materials for the synthesis of silicone polymers across various fields such as children’s products, cosmetics, electronic products, and food contact materials, and their conclusive evidence of human hazards has prompted regulatory concerns and restrictions. Thus, manufacturers have responded by reducing the amount of D4, D5, and D6 in products to comply with the production criteria. The well-documented persistence and high bioaccumulation of D4, D5, and D6 have long attracted worldwide attention. Canada is the first country to pay special consideration to the toxicity of DMCs, after which ECHA classified D4, D5, and D6 as SCHV substances and proposed to list them as POPs. The European Union also submitted a notification G/TBT/N/EU/989 to the World Trade Organization (WTO), which, in brief, restricted the application of these substances in textiles, leather, and rinse-off cosmetics in an attempt to minimize their emission [37]. On the other hand, D7, D8, and D9 are mainly intermediate by-products of the synthesis of silicone polymers, attracting less regulatory attention, and their use remains unrestricted. In this study, the compositional profiles of DMCs varied considerably across diverse samples, which, in addition to distinct restrictions, may be due to disparities in product manufacturing processes. However, until now, there is limited information on the toxicity of D7, D8, and D9. Indeed, D7, D8, and D9 have similar chemical structures to D4, D5, and D6, so they are speculated to have similar environmental and human hazardous effects. Considering the findings in this investigation, the proportion of D7, D8, and D9 within the ∑DMCs of the selected samples cannot be neglected, especially in silicone rubber samples. Hence, their presence in mobile smart terminal devices warrants careful consideration and further investigation.

### 3.3. Environmental Emissions of DMCs from Silicone Rubber

The estimated amount of ∑DMCs released into the environment in China from silicone rubber used in mobile smart terminal devices was 5381.56 tons per year. The silicone rubber samples selected in this study comprised ear caps, watch bands, and case covers, which were replaced frequently and discarded as household waste due to their low price and a wide variety of styles. Substances like DMCs in these mobile smart terminal devices will eventually enter the environment through processes such as e-waste dismantling [46] and thermo-treatment [47]. Previous studies have reported half-lives exceeding 4 days for D6 and up to 10–30 days for D3–D5, facilitating their long-range environmental transfer [55,61]. Notably, airborne DMCs have an extremely high potential to form secondary organic aerosols [62], with inhalation representing the primary route of human exposure to interior airborne DMCs [63]. Furthermore, a study conducted in the Bohai Sea, China, demonstrated the biotrophic magnification of D4–D7 in aquatic food webs [64], thereby elevating the concentrations of DMCs accessible to humans via dietary intake. Collectively, DMCs released into the environment pose a threat to human health through multiple exposure routes. Unfortunately, it is known that 80% of the discarded mobile smart terminal devices are not properly recycled [50]. Thus, there is considerable merit in investigating and recognizing the environmental hazards of DMCs, not only in silicone rubber materials, but also in other device components. It is advisable to explore alternative materials such as metal or woven watchbands instead of silicone rubber ones, and sponge ear caps rather than silicone ear caps. Additionally, it is recommended for manufacturers of mobile smart terminal products to optimize the vulcanization process in silicone rubber production to diminish the content of DMCs.

Nevertheless, this study still has several limitations. First, while the chosen samples selected in our study, namely silicone rubber, plastics, and adhesives, were identified as the most representative and high-risk sources of DMCs, the sample sizes remain modest, with only 22, 18, and 12 specimens, respectively. Second, it is difficult to accurately calculate the environmental emissions of DMCs due to the varied types and proportions of adhesive and plastics used in different types of mobile smart terminal devices and further refinement of experimental methodologies is needed to ensure the validity and accuracy of the data. Third, there are also gaps in the toxicological studies and ecological hazards of D7, D8, and D9, which pose a significant obstacle to elucidating the mechanisms by which DMCs induce deleterious effects. However, this is the first study to provide valid information on the levels of DMCs in mobile smart terminal devices and a stable quantitative method. Future studies could encompass large-scale product categories and expanded sample sizes, as well as incorporate degradation byproducts of DMCs, such as those resulting from UV irradiation or oxidation, to comprehensively scrutinize the environmental and human health implications of DMCs. Toxicological studies of D7-D9 and its congeners could facilitate the development of mathematical models, elucidating the metabolism of DMCs in humans, enhancing our microscopic comprehension of the characteristics of DMCs. Furthermore, the quantification of DMCs in commodities, food, environmental media, and human body fluids (e.g., urine, blood, and breast milk) will allow an effective estimation of DMCs releases and their risk to humans, which can inform the establishment of more precise regulatory thresholds for the application of DMCs.

## 4. Conclusions

The DMCs were widely detected in over 95% of silicone materials used in mobile smart terminal devices. D7, D8, and D9 were dominant in silicone rubbers, while D4, D5, and D6 were predominant in adhesives, collectively constituting over 80% and 60% of ∑DMCs concentrations, respectively. The estimated annual release of ∑DMCs into the environment in China from silicone rubber used in mobile smart terminal devices exceeds 5000 tons. Further studies are needed on the toxicological effects of D7–D9 and the possible degradation byproducts of DMCs. Focus should also be placed on the fate of DMCs in the environment, their internal and external exposure to humans, and risk assessments to clarify their threat to human health and the regulation of these chemicals.

## Figures and Tables

**Table 1:** Information of targeted DMCs.

Name	Abbreviation	CAS-No.	Boiling Points	Molar Weight
Hexamethylcyclotrisiloxane	D3	541-05-9	134 °C	222.47
Octamethylcyclotetrasiloxane	D4	556-67-2	175–176 °C	296.62
Decamethylcyclopentasiloxane	D5	541-02-6	205 °C	370.72
Dodecamethylcyclohexasiloxane	D6	540-97-6	245 °C	444.93
Tetradecamethylcycloheptasiloxane	D7	107-50-6	337 °C	519.08
Hexadecamethylcyclooctasiloxane	D8	556-68-3	290 °C	593.23
Octadecamethylcyclononasiloxane	D9	556-71-8	416 °C	667.39

**Table 2:** Concentrations of DMCs in silicone rubber, plastic, and adhesive samples (mg/kg).

Samples	Statistics	D3	D4	D5	D6	D7	D8	D9	D4+D5+D6	D7+D8+D9	∑DMCs
Silicone rubber (*n* = 22)	DR (%)	9	59	95.5	95.5	95.5	91	91	95.5	95.5	95.5
	Mean	0.53	9.91	66.3	136.9	159.3	171.8	159.0	212.9	490.0	703.0
	Median	0.50	1.55	14.5	78.9	180.1	158.2	132.5	86.0	481.5	802.2
	Range	<LOQ-1.1	<LOQ-63	<LOQ-335	<LOQ-422	<LOQ-479	<LOQ-481	<LOQ-438.9	<LOQ-820	<LOQ-1331	<LOQ-1788
Plastics (*n* = 18)	DR (%)	61.1	61.1	50	38.9	44.4	50	50	61.1	50	66.7
	Mean	22.1	19.0	9.36	5.32	4.46	4.53	3.69	33.1	12.2	66.9
	Median	2.95	2.15	0.50	0.50	0.50	0.75	1.65	2.40	2.40	8.25
	Range	<LOQ-279	<LOQ-181	<LOQ-86	<LOQ-42.5	<LOQ-27.5	<LOQ-33.6	<LOQ-26.4	<LOQ-309.5	<LOQ-87.5	<LOQ-631.6
Adhesive (*n* = 12)	DR (%)	41.7	50	100	100	91.7	75	50	100	91.7	100
	Mean	2.11	5.33	28.9	43.8	38.2	41.9	48.4	78.5	129.0	208.6
	Median	0.50	0.95	2.35	7.35	2.10	1.20	0.90	12.1	5.0	21.5
	Range	<LOQ-10.3	<LOQ-27.7	1–119	1.8–160	<LOQ-153	<LOQ-169	<LOQ-204	3.7–289.6	<LOQ-515	6.3–807.9
All (*n* = 52)	DR (%)	34.6	57.7	80.8	76.9	76.9	73.1	67.3	84.6	78.8	86.5
	Mean	8.52	12.0	38.0	79.9	77.8	83.9	79.7	119.7	241.3	368.7
	Median	0.50	1.55	6.60	11.0	7.35	8.10	7.55	33.3	23.2	70.8
	Range	<LOQ-279	<LOQ-181	<LOQ-335	<LOQ-422	<LOQ-479	<LOQ-481	<LOQ-438.9	<LOQ-820	<LOQ-1331	<LOQ-1788

∑DMCs: the sum concentration of D3, D4, D5, D6, D7, D8, and D9; DR: detection rates, $DR=\left(\frac{number\;of\;samples\;with\;DMCs\;detected}{total\;number\;of\;samples}\right)\times 100\%$.

**Table 3:** The levels of DMCs in different samples from various regions.

Region	Samples	Sample Size	Statistics	D3	D4	D5	D6	D7	D8	D9	Units	References
USA	Silicone nipples	22	DR (%)	-	100	100	100	-	-	-		Zhang et al., 2012 [9]
			Range	-	0.6–49	0.6–269	0.3–108	-	-	-	mg/kg	
Canada	Cosmetic products	252	DR (%)	0.79	4.8	14	9.1	-	-	-		Wang et al., 2009 [7]
China	Soft rubber toys	44	DR (%)	-	100	91	100	-	-	-		Xu et al., 2017 [58]
			Median	-	0.062	0.086	0.101	-	-	-	μg/g	
	Pacifiers	74	DR (%)	-	100	100	100	-	-	-		
			Median	-	0.107	0.924	1.46	-	-	-	μg/g	
	Hard toys	40	DR (%)	-	10	50	40	-	-	-		
			Median	-	<0.6 × 10^−3^	<0.45 × 10^−3^	<0.7 × 10^−3^	-	-	-	μg/g	
Europe	Cosmetics and PCPs	51	Median	-	0.011	25.7	0.64	-	-	-	mg/g	Dudzina et al., 2014 [4]
Germany	Bakeware products	4	Mean	-	505	284	753	1265	1127	1325	mg/kg	Fromme et al., 2019 [5]
Shanghai, China	PCPs	158	Median	-	0.29	1.70	0.66	0.06	-	-	μg/g	Lu et al., 2011 [59]

## Data Availability

Data are available from the corresponding author by request.

## Supplementary Materials

The following supporting information can be downloaded at: https://www.mdpi.com/article/10.3390/toxics12040287/s1, Table S1: Retention times, qualitative ions, quantitative ions and ion-pair ratios of DMCs; Table S2: The standard curve, regression coefficients (*R*^2^), and limit of quantitation of each analyte.

## References

[1] Horii Y., Minomo K., Lam J.C.W., Yamashita N. (2022). Spatial Distribution and Accumulation Profiles of Volatile Methylsiloxanes in Tokyo Bay, Japan: Mass Loadings and Historical Trends. Sci. Total Environ..

[2] Molinier B., Arata C., Katz E., Lunderberg D., Liu Y., Misztal P., Nazaroff W., Goldstein A. (2022). Volatile Methyl Siloxanes and Other Organosilicon Compounds in Residential Air. Environ. Sci. Technol..

[3] Gerhards R., Seston R.M., Kozerski G.E., McNett D.A., Boehmer T., Durham J.A., Xu S. (2022). Basic Considerations to Minimize Bias in Collection and Analysis of Volatile Methyl Siloxanes in Environmental Samples. Sci. Total Environ..

[4] Dudzina T., von Goetz N., Bogdal C., Biesterbos J., Hungerbühler K. (2014). Concentrations of Cyclic Volatile Methylsiloxanes in European Cosmetics and Personal Care Products: Prerequisite for Human and Environmental Exposure Assessment. Environ. Int..

[5] Fromme H., Witte M., Fembacher L., Gruber L., Hagl T., Smolic S., Fiedler D., Sysoltseva M., Schober W. (2019). Siloxane in Baking Moulds, Emission to Indoor Air and Migration to Food during Baking with an Electric Oven. Environ. Int..

[6] Varaprath S., Stutts D.H., Kozerski G.E. (2006). A Primer on the Analytical Aspects of Silicones at Trace Levels-Challenges and Artifacts—A Review. Silicon Chem..

[7] Wang R., Moody R., Koniecki D., Zhu J. (2009). Low Molecular Weight Cyclic Volatile Methylsiloxanes in Cosmetic Products Sold in Canada: Implication for Dermal Exposure. Environ. Int..

[8] Zammarano M., Cazzetta V., Nazaré S., Shields J.R., Kim Y.S., Hoffman K.M., Maffezzoli A., Davis R. (2016). Smoldering and Flame Resistant Textiles via Conformal Barrier Formation. Adv. Mater. Interfaces.

[9] Zhang K., Wong J.W., Begley T.H., Hayward D.G., Limm W. (2012). Determination of Siloxanes in Silicone Products and Potential Migration to Milk, Formula and Liquid Simulants. Food Addit. Contam. Part. A Chem. Anal. Control Expo. Risk Assess..

[10] Horii Y., Ohtsuka N., Minomo K., Takemine S., Motegi M., Hara M. (2021). Distribution Characteristics of Methylsiloxanes in Atmospheric Environment of Saitama, Japan: Diurnal and Seasonal Variations and Emission Source Apportionment. Sci. Total Environ..

[11] Guo J., Zhou Y., Zhang B., Zhang J. (2019). Distribution and Evaluation of the Fate of Cyclic Volatile Methyl Siloxanes in the Largest Lake of Southwest China. Sci. Total Environ..

[12] Nu Nguyen H.M., Khieu H.T., Ta N.A., Le H.Q., Nguyen T.Q., Do T.Q., Hoang A.Q., Kannan K., Tran T.M. (2021). Distribution of Cyclic Volatile Methylsiloxanes in Drinking Water, Tap Water, Surface Water, and Wastewater in Hanoi, Vietnam. Environ. Pollut..

[13] Zhang Y., Shen M., Tian Y., Zeng G. (2018). Cyclic Volatile Methylsiloxanes in Sediment, Soil, and Surface Water from Dongting Lake, China. J. Soil. Sediment..

[14] Wang D.-G., Alaee M., Steer H., Tait T., Williams Z., Brimble S., Svoboda L., Barresi E., DeJong M., Schachtschneider J. (2013). Determination of Cyclic Volatile Methylsiloxanes in Water, Sediment, Soil, Biota, and Biosolid Using Large-Volume Injection-Gas Chromatography-Mass Spectrometry. Chemosphere.

[15] Tran T., Abualnaja K., Asimakopoulos A., Covaci A., Gevao B., Johnson-Restrepo B., Kumosani T., Malarvannan G., Minh T., Moon H. (2015). A Survey of Cyclic and Linear Siloxanes in Indoor Dust and Their Implications for Human Exposures in Twelve Countries. Environ. Int..

[16] Zhu Y., Tang Z., He Y., Wang F., Lyu Y. (2023). Occurrence of Methylsiloxanes in Indoor Store Dust in China and Potential Human Exposure. Environ. Res..

[17] Wang D., de Solla S., Lebeuf M., Bisbicos T., Barrett G., Alaee M. (2017). Determination of Linear and Cyclic Volatile Methylsiloxanes in Blood of Turtles, Cormorants, and Seals from Canada. Sci. Total Environ..

[18] Zhi L., Xu L., He X., Zhang C., Cai Y. (2019). Distribution of Methylsiloxanes in Benthic Mollusks from the Chinese Bohai Sea. J. Environ. Sci..

[19] Guo J., Zhou Y., Wang Y., Zhang B., Zhang J. (2021). Assessment of Internal Exposure to Methylsiloxanes in Children and Associated Non-Dietary Exposure Risk. Environ. Int..

[20] Krenczkowska D., Mojsiewicz-Pienkowska K., Wielgomas B., Bazar D., Jankowski Z. (2020). Ex Vivo Human Skin Is Not a Barrier for Cyclic Siloxanes (Cyclic Silicones): Evidence of Diffusion, Bioaccumulation, and Risk of Dermal Absorption Using a New Validated GC-FID Procedure. Pharmaceutics.

[21] Niu H., Su X., Li Q., Zhao J., Hou M., Dong S., Yan X., Sun J., Feng J. (2023). Dimethylsiloxanes in Dust from Nine Indoor Microenvironments of Henan Province: Occurrence and Human Exposure Assessment. Sci. Total Environ..

[22] Sun H., Li D., Xu L., Qiu C., Wang S., Liu N., Sun L. (2022). Research Progress on the Distribution, Behavior and Effects of Cyclic Volatile Methylsiloxanes in Organisms. J. Soil. Sediment..

[23] Kumari K., Singh A., Marathe D. (2023). Cyclic Volatile Methyl Siloxanes (D4, D5, and D6) as the Emerging Pollutants in Environment: Environmental Distribution, Fate, and Toxicological Assessments. Environ. Sci. Pollut. Res..

[24] Franzen A., Greene T., Van Landingham C., Gentry R. (2017). Toxicology of Octamethylcyclotetrasiloxane (D4). Toxicol. Lett..

[25] Zhang J., Falany J., Xie X., Falany C. (2000). Induction of Rat Hepatic Drug Metabolizing Enzymes by Dimethylcyclosiloxanes. Chem. Biol. Interact..

[26] Lee J., Kim K., Park S.-M., Kwon J.-S., Jeung E.-B. (2023). Effects of Decamethylcyclopentasiloxane on Reproductive Systems in Female Rats. Toxics.

[27] Siddiqui W.H., Stump D.G., Plotzke K.P., Holson J.F., Meeks R.G. (2007). A Two-Generation Reproductive Toxicity Study of Octamethylcyclotetrasiloxane (D4) in Rats Exposed by Whole-Body Vapor Inhalation. Reprod. Toxicol..

[28] Tran D.N., Park S.-M., Jung E.-M., Jeung E.-B. (2021). Prenatal Octamethylcyclotetrasiloxane Exposure Impaired Proliferation of Neuronal Progenitor, Leading to Motor, Cognition, Social and Behavioral Functions. Int. J. Mol. Sci..

[29] Hossain M.M., Yuan Y., Huang H., Wang Z., Baki M.A., Dai Z., Rizwan M., Xiong S., Cao M., Tu S. (2021). Exposure to Dodecamethylcyclohexasiloxane (D6) Affects the Antioxidant Response and Gene Expression of *Procambarus clarkii*. Sustainability.

[30] Jean P.A., Plotzke K.P. (2017). Chronic Toxicity and Oncogenicity of Octamethylcyclotetrasiloxane (D4) in the Fischer 344 Rat. Toxicol. Lett..

[31] Young L.J., Morfeld P. (2016). Statistical Considerations for a Chronic Bioassay Study: Exposure to Decamethylcyclopentasiloxane (D5) and Incidence of Uterine Endometrial Adenocarcinomas in a 2-Year Inhalation Study with Fischer Rats. Regul. Toxicol. Pharmacol..

[32] Farasani A., Darbre P.D. (2017). Exposure to Cyclic Volatile Methylsiloxanes (cVMS) Causes Anchorage-Independent Growth and Reduction of BRCA1 in Non-Transformed Human Breast Epithelial Cells. J. Appl. Toxicol..

[33] Zhang B., Zhou Y., Guo J. (2023). Association of Volatile Methylsiloxanes Exposure with Non-Alcoholic Fatty Liver Disease among Chinese Adults. Environ. Pollut..

[34] Cantu M., Gobas F. (2021). Bioaccumulation of Dodecamethylcyclohexasiloxane (D6) in Fish. Chemosphere.

[35] Wang D.-G., Norwood W., Alaee M., Byer J.D., Brimble S. (2013). Review of Recent Advances in Research on the Toxicity, Detection, Occurrence and Fate of Cyclic Volatile Methyl Siloxanes in the Environment. Chemosphere.

[36] ECHA Candidate List of Substances of Very High Concern for Authorisation. https://echa.europa.eu/candidate-list-table.

[37] ECHA List of Substances Proposed as POPs. https://echa.europa.eu/list-of-substances-proposed-as-pops/-/dislist/details/0b0236e184f17c3e.

[38] ACMI (2023). 2023 China Silicone Series Product Market Report Was Released Grandly.

[39] Arshad, Nekahi A., McMeekin S.G., Farzaneh M. (2018). Measurement of Surface Resistance of Silicone Rubber Sheets under Polluted and Dry Band Conditions. Electr. Eng..

[40] Li G., Gong J.M., Tan J.Z., Zhu D.S., Jia W.H., Lu X.J. (2019). Acidic-Thermal Ageing Effect on Compression Stress Relaxation of Silicone Rubber. Strength. Mater..

[41] Mollah M.S.I., Kwon Y.-D., Islam M.M., Seo D.-W., Jang H.-H., Lim Y.-D., Lee D.-K., Kim W.-G. (2012). Synthesis and Characterization of Polycarbonates Containing Terminal and Chain Interior Siloxane. Polym. Bull..

[42] Yuan D., Cai X. (2013). Synthesis of a Silicon-Containing Flame Retardant and Its Synergistic Effect with Potassium-4-(Phenylsulfonyl)Benzenesulfonate (KSS) in Polycarbonate (PC). Chin. J. Polym. Sci..

[43] Antosik A.K., Makuch E., Gziut K. (2022). Influence of Modified Attapulgite on Silicone Pressure-Sensitive Adhesives Properties. J. Polym. Res..

[44] Antosik A.K., Weisbrodt M., Mozelewska K., Czech Z., Piątek-Hnat M. (2020). Impact of Environmental Conditions on Silicone Pressure-Sensitive Adhesives. Polym. Bull..

[45] Storozhenko P.A., Minas’yan R.M., Polivanov A.N., Nikitushkin I.V., Minas’yan O.I. (2017). New Thermally Conductive Silicone Adhesive Sealants. Polym. Sci. Ser. D.

[46] He X., Xu L., Zhang C., Cai Y. (2016). Pollution Characteristics of Methyl Siloxanes in Soil from an Electronic Waste(e-Waste) Dismantling Area in Taizhou, China. J. Soil. Sediment..

[47] Xu L., Huang Z., Zhang Q., Xiang X., Zhang S., Cai Y. (2020). Methylsiloxanes and Their Brominated Products in One E-Waste Recycling Area in China: Emission, Environmental Distribution, and Elimination. Environ. Sci. Technol..

[48] ACMI (2022). Adhesives Market Report.

[49] Amghar N., Moreno V., Sánchez-Jiménez P.E., Perejón A., Pérez-Maqueda L.A. (2024). Ca-Based Materials Derived from Calcined Cigarette Butts for CO_2_ Capture and Thermochemical Energy Storage. J. Environ. Sci..

[50] Forti V., Bald C.P., Kuehr R., Bel G. The Global E-Waste Monitor 2020. E-Waste Monitor. https://ewastemonitor.info/gem-2020/.

[51] Fromme H., Cequier E., Kim J., Hanssen L., Hilger B., Thomsen C., Chang Y., Völkel W. (2015). Persistent and Emerging Pollutants in the Blood of German Adults: Occurrence of Dechloranes, Polychlorinated Naphthalenes, and Siloxanes. Environ. Int..

[52] Hanssen L., Warner N., Braathen T., Odland J., Lund E., Nieboer E., Sandanger T. (2013). Plasma Concentrations of Cyclic Volatile Methylsiloxanes (cVMS) in Pregnant and Postmenopausal Norwegian Women and Self-Reported Use of Personal Care Products (PCPs). Environ. Int..

[53] Xu L., Shi Y., Liu N., Cai Y. (2015). Methyl Siloxanes in Environmental Matrices and Human Plasma/Fat from Both General Industries and Residential Areas in China. Sci. Total Environ..

[54] ECHA Background Document to the Opinion on the Annex XV Dossier Proposing Restrictions on Octamethylcyclotetrasiloxane (D4) and Decamethylcyclopentasiloxane (D5), 2016. https://echa.europa.eu/documents/10162/fefaa3a2-ffc0-4b74-4ec8-3c869d4adae7.

[55] Xiao R., Zammit I., Wei Z., Hu W.-P., MacLeod M., Spinney R. (2015). Kinetics and Mechanism of the Oxidation of Cyclic Methylsiloxanes by Hydroxyl Radical in the Gas Phase: An Experimental and Theoretical Study. Environ. Sci. Technol..

[56] IDC IDC Media Center. IDC: The Premier Global Market Intelligence Company. https://www.idc.com/about/press.

[57] Horii Y., Kannan K. (2008). Survey of Organosilicone Compounds, Including Cyclic and Linear Siloxanes, in Personal-Care and Household Products. Arch. Environ. Contam. Toxicol..

[58] Xu L., Zhi L., Cai Y. (2017). Methylsiloxanes in Children Silicone-Containing Products from China: Profiles, Leaching, and Children Exposure. Environ. Int..

[59] Lu Y., Yuan T., Wang W., Kannan K. (2011). Concentrations and Assessment of Exposure to Siloxanes and Synthetic Musks in Personal Care Products from China. Environ. Pollut..

[60] Helling R., Mieth A., Altmann S., Simat T.J. (2009). Determination of the Overall Migration from Silicone Baking Moulds into Simulants and Food Using 1H-NMR Techniques. Food Addit. Contam. Part. A Chem. Anal. Control Expo. Risk Assess..

[61] Kim J., Xu S. (2017). Quantitative Structure-reactivity Relationships of Hydroxyl Radical Rate Constants for Linear and Cyclic Volatile Methylsiloxanes. Environ. Toxicol. Chem..

[62] Xu J., Harrison R.M., Song C., Hou S., Wei L., Fu P., Li H., Li W., Shi Z. (2022). PM2.5-Bound Silicon-Containing Secondary Organic Aerosols (Si-SOA) in Beijing Ambient Air. Chemosphere.

[63] Tran T.M., Hoang A.Q., Le S.T., Minh T.B., Kannan K. (2019). A Review of Contamination Status, Emission Sources, and Human Exposure to Volatile Methyl Siloxanes (VMSs) in Indoor Environments. Sci. Total Environ..

[64] Cui S., Fu Q., An L., Yu T., Zhang F., Gao S., Liu D., Jia H. (2019). Trophic Transfer of Cyclic Methyl Siloxanes in the Marine Food Web in the Bohai Sea, China. Ecotoxicol. Environ. Saf..

