# Supplementary material for: Dimethylcyclosiloxanes in Mobile Smart Terminal Devices: Concentrations, Distributions, Profiles, and Environmental Emissions
Source: Toxics. 2024 Apr 13;12(4):287. doi: 10.3390/toxics12040287 (PMC11053745; doi:10.3390/toxics12040287)
Supplement: Supplementary file 1 [file toxics-12-00287-s001.zip › toxics-2940619-supplementary.pdf]

Supplementary Materials

Dimethylcyclosiloxanes in mobile smart terminal devices: concentrations, distributions, profiles, and environmental emissions

Yuanna Xing [a,c], Yiming, Ge [b], Shaoyou Lu [b], Xianzhi Peng [a,c*]

[a] Guangzhou Institute of Geochemistry, Chinese Academy of Sciences, Guangzhou, 510640, China

[b] School of Public Health (Shenzhen), Shenzhen Campus of SunYat-sen University, Shenzhen, 518107, China

[c] University of Chinese Academy of Sciences, Beijing, 100049, China

[*]**Corresponding author:** pengx@gig.ac.cn (X Peng)

Table S1 Retention times, qualitative ions, quantitative ions and ion-pair ratios of DMCs

Analytes	Retention time (min)	Qualitative ion (m/z)	Quantitative ion (m/z)	Ion-pair ratio (%)
D3	5.159	207	96	11.74
D4	7.540	281	265	13.64
D5	9.038	355	267	98
D6	10.990	341	429	43.8
D7	12.480	281	327	42.7
D8	13.800	355	281	29.2
D9	14.934	429	355	54.7

Table S2 The standard curve, regression coefficients (R^2), and limit of quantitation of each analyte

Analytes	Standard curve	R^2	LOQ (mg/kg)
D3	y=46874x+71732	0.9993	1
D4	y=449965x+41868	0.9994	1
D5	y=208802x+14271	0.9994	1
D6	y=285114x+16653	0.9996	1
D7	y=233099x+12163	0.9997	1
D8	y=236203x+15366	0.9997	1
D9	y=179769x+11016	0.9997	1
